# CT-guided percutaneous biopsy of spinal lesions

**DOI:** 10.2349/biij.2.3.e25

**Published:** 2006-07-01

**Authors:** WCG Peh

**Affiliations:** Programme Office, Singapore Health Services, Singapore

**Keywords:** Biopsy, computed tomography, intervention, percutaneous biopsy, spine, vertebral lesions

## Abstract

Accurate diagnosis of spine lesions is important for its successful management. Imaging–guided percutaneous biopsy is gaining increasing acceptance as a means for obtaining tissue for diagnosis. Most biopsies can be rapidly performed under local anaesthesia, with little patient discomfort and improved safety. Spinal anatomy is, however, complex with many adjacent vital structures. Good knowledge of anatomy and precise needling technique is, therefore, important. Today, biopsy of spinal lesions is best performed under computed tomography (CT) fluoroscopic guidance. Indications for imaging-guided biopsy include confirming metastasis in a patient with a known primary tumour, determining the nature of a solitary bone lesion, excluding malignancy in vertebral body compression, and investigating for infection. Among the various issues to be considered are site of lesion, location of adjacent vital structures, approach, and type and size of needle. Complications are rare, particularly when a meticulous technique is applied. In summary, CT-guided percutaneous biopsy is a safe and an effective technique for the evaluation of spinal lesions and useful in planning therapy.

## INTRODUCTION

Biopsy entails the removal of tissue from a living body with the aim of establishing a precise diagnosis, usually by microscopic examination or culture. It can be performed during surgery (open biopsy) or percutaneously (closed biopsy). Percutaneous biopsy of bony lesions may be performed under the guidance of a variety of imaging modalities, such as, fluoroscopy [1-4], computed tomography (CT) [5-19], ultrasonography [20-24], and magnetic resonance (MR) imaging [25-28]. Open biopsy is a major surgical procedure that is associated with morbidity and a host of complications. There are several advantages of imaging-guided biopsy, including avoidance of overnight hospital stay, cost and time saving, earlier commencement of radiation therapy, biopsy of surgically-inaccessible sites, and lower morbidity. The last mentioned advantage includes avoidance of general anaesthetic-related complications, less risk of postoperative wound infection, and decreased likelihood of development of pathological fracture [29-31].

Imaging- guided biopsy of bony lesions is a safe procedure, with development of major complications being rare. Reported accuracy ranges from 68% to 97% [1-4,7,8,10,12,14,16,17,19,21,28,29,32-38]. Fluoroscopy is most often used to guide biopsy of lesions in long bones. Ultrasonography can be used for lesions on or near the surface of bone and, particularly, if there is an associated soft tissue mass or an adjacent cortical destruction [20-24]. MR imaging has been advocated for biopsy of musculoskeletal lesions, particularly for those that are not clearly visible on fluoroscopy or CT [25-28]. The choice of imaging guidance modality to be employed is determined by individual operator preference and the availability of equipment and facilities, e.g., open MR magnet and MR-compatible accessories.

**Figure 1:**
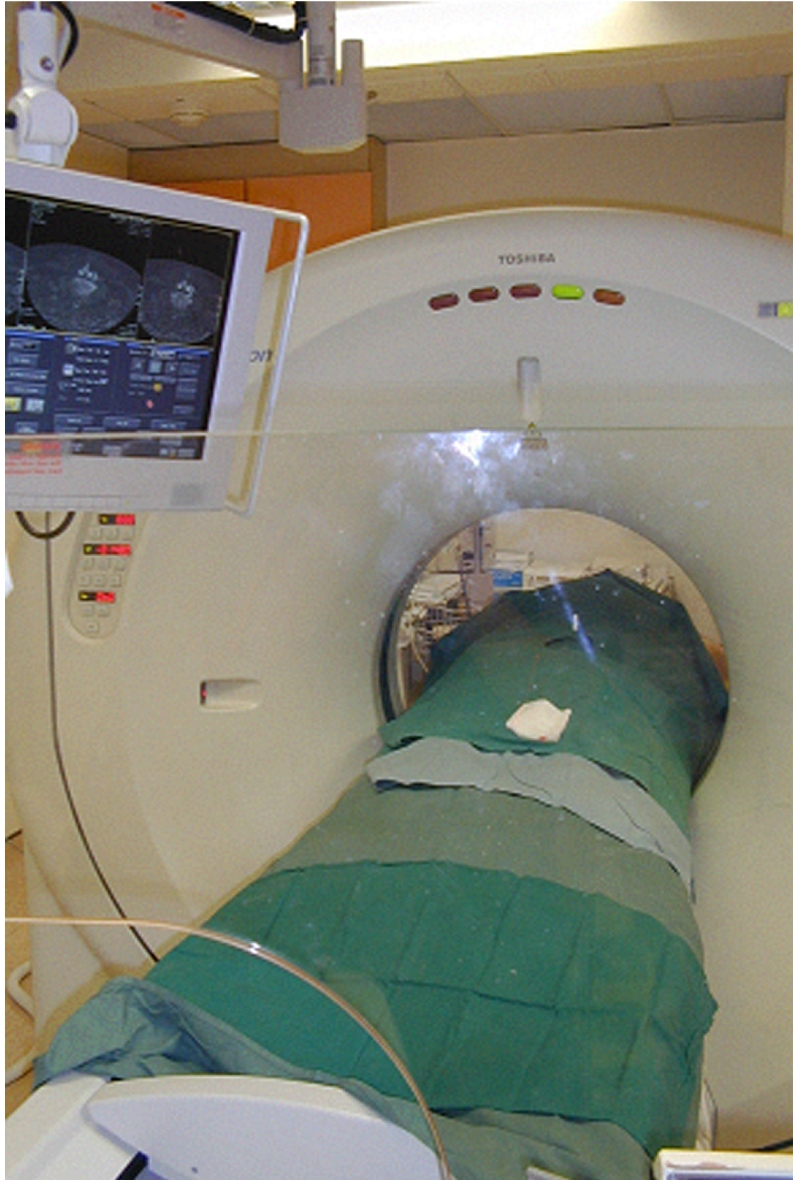
Photograph of a CT fluoroscopy unit. The radiologist stands behind the lead glass shield in the foreground and moves the CT couch-top either manually or via remote control. The CT images are observed on the monitor.

Although the use of fluoroscopy [3,4], ultrasonography [21], and MR imaging [28] has been described, CT is currently the modality of choice for guiding biopsy of lesions of the spine [5,6,8,10-12,14-19]. Ultrasonography has been advocated for guiding biopsy of cervical spine lesions, and in the thoracic and lumbosacral regions its use is limited to lesions affecting the posterior elements [21]. Compared with fluoroscopy, CT more precisely shows the needle position and is potentially safer as there is less likelihood of injury to adjacent structures, such as, major vessels and nerve roots. With CT fluoroscopy, near real-time imaging is possible (Figure 1). The radiation dosage in CT fluoroscopy is also relatively small, compared with conventional diagnostic CT [39]. This review highlights the utility of CT fluoroscopy to guide percutaneous biopsy of spinal lesions.

## GENERAL PRINCIPLES AND PREPARATION

A well-planned and executed biopsy is essential for accurate diagnosis and enables appropriate treatment. For a suspected primary tumour, biopsy should be performed at centres that specialise in the treatment of bone tumours. At these centres, joint management decisions are taken by a multidisciplinary team that includes surgeons, oncologists, pathologists, and radiologists. All imaging, particularly MR imaging, should be completed prior to biopsy. The radiologist should review the clinical data and all imaging studies to ensure that a biopsy is indicated and to determine the most appropriate biopsy site. Radiographs provide an overview of the lesion and its characteristics, and they are probably the best single modality for providing a diagnosis or short list of differential diagnoses. In complex-shaped bones, such as, the spine, CT aids in showing lesion details and the presence of associated calcification. Bone scintigraphy helps determine the extent and distribution of the disease. MR imaging is best for defining the local extent of the disease, particularly for radiographically-occult or subtle lesions, marrow involvement, and associated soft tissue components [40].

The biopsy route should be planned such that it does not compromise the neurovascular bundles, and uninvolved tissue compartments should not be crossed. The biopsy should be done in consultation with the surgeon who will be performing the definitive surgery and ideally also with the pathologist who will be examining the specimen. The radiologist should aim to obtain at least three specimens during biopsy, particularly if a cytotechnologist is not present during the procedure to confirm adequacy of the biopsy specimen. Anticoagulants should be discontinued prior to biopsy, and for assessment of infection antibiotics should be stopped at least 48 hours before the biopsy. Other factors to be considered prior to biopsy are blood tests for coagulopathy (prothrombin time, activated partial thromboplastin time, international normalised ratio), platelet counts, need for sedation or anti-anxiety premedication, and written informed consent [40].

## INDICATIONS

**Figure 2:**
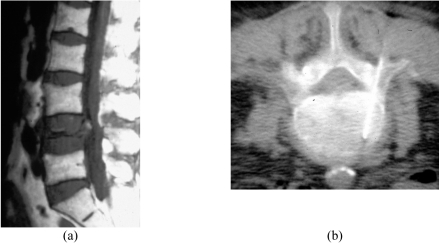
Severe L4 vertebral body compression in a 77-year-old woman. (a) Sagittal SE T1-W MR image shows severe compression of the L4 vertebral body. (b) Axial CT fluoroscopic image taken with the patient lying prone shows the Ostycut needle inserted into the right side of the L4 vertebral body via the transpedicular route.

**Figure 3:**
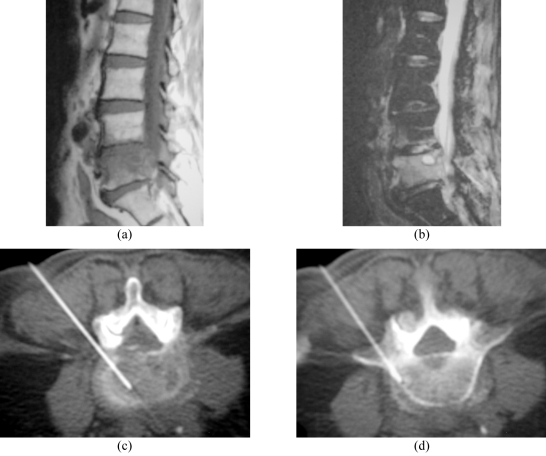
L4/5 infective spondylodiscitis in a 64-year-old man. (a) Sagittal SE T1-W MR image shows an ill-defined hypointense lesion involving most of the L5 vertebral body, with irregularity of the superior endplate. (b) Sagittal fat-suppressed FSE T2-W MR image shows hyperintense signal in the L5 vertebral body and the L4/5 disc, with anterior subligamentous and posterior extradural extension. Appearances are typical of L5 vertebral osteomyelitis with L4/5 discitis. Axial CT fluoroscopic images taken with the patient lying prone show the Ostycut needle inserted into the (c) L4/5 disc and (d) L5 superior subchondral vertebral body via a left posterolateral paravertebral approach.

Indications for biopsy of spinal lesions include [14,40-43]:

- Confirm or exclude metastasis in a patient with a known primary tumour.
- Determine the nature of a solitary bone lesion with non-specific imaging findings.
- Exclude malignancy in vertebral body compression, especially metastases or myeloma (Figure 2).
- Evaluation for tumour recurrence.
- Investigation for infection to confirm diagnosis and to obtain sample of organism, e.g., discitis or osteomyelitis (Figure 3).

## CONTRAINDICATIONS

Contraindications include [40-43]:

- Bleeding diathesis.
- Decreased platelet count (<50,000/mm^3^).
- Suspected vascular lesion in the thoracic vertebra. Haemorrhage leading to cord compression may occur.
- Infected soft tissues surrounding bony lesion to be biopsied, particularly when a non-infective lesion is suspected.
- Inaccessible sites, e.g., C1 and odontoid lesions.
- Uncooperative patient. General anaesthesia may be required.

## TECHNIQUE AND EQUIPMENT

During CT fluoroscopy the patient is positioned to facilitate needle access to the lesion and, ideally, also to ensure as much comfort as possible. For biopsy of thoracic and lumbosacral spine lesions, the patient usually lies in a prone position. For the cervical spine, patients may be placed prone for lesions located in the pedicles and posterior elements. For anteriorly-located cervical spine lesions, the patient lies in a supine position. Sometimes, the patient may have to be placed in a lateral decubitus, semi-prone or semi-supine position, to ensure patient comfort and to minimise patient movement. Although the radiation dosage in CT fluoroscopy is relatively small, compared with conventional diagnostic CT [39], care should still be taken to minimise irradiation to both the patient and the operator. Adopting very short bursts of intermittent CT fluoroscopic screening, using “last image hold” to study the needle position on the monitor, and saving a few representative CT fluoroscopic images from the monitor for reporting purposes will help reduce radiation dosage to the patient. It is possible for the operator to avoid radiation exposure altogether. The operator stands behind a lead glass shield in the CT suite and moves the CT couch-top via remote control. My own preference is to move the couch-top manually, screen intermittently only while standing behind the lead glass shield, and make several fine adjustments to the needle position after studying the images on the monitor, until the needle tip is in the correct position (Figure 1).

**Figure 4:**
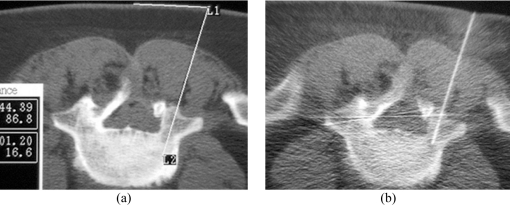
Planning of the approach and the needle route. (a) Preliminary axial CT image shows measurement of the distance between the proposed puncture site and the midline, as well as the depth and the angulation to targeted biopsy site in L5 vertebral body. (b) Axial CT fluoroscopic image taken with the patient lying prone shows the Ostycut needle inserted into the same vertebral body via the right transpedicular route.

The patient’s vital signs should be continuously monitored during the procedure. Following preliminary axial CT scanning, the most appropriate slice is selected to plan the most ideal route for directing the needle into the lesion (Figure 4). Generally, if there are multiple lesions, the largest and most superficial lesion is chosen. Any soft tissue mass related to the bony lesion should also be biopsied. For suspected discitis, in addition to the disc, the adjacent subchondral bone should also be biopsied as this is the site of origin of haematogeneous spondylodiscitis [19] (Figure 3). In planning the needle route, vital anatomical structures, e.g., major blood vessels, nerves, peritoneal cavity, and spinal canal and its contents, should be avoided. In the cervical spine, important structures to avoid include the trachea, oesophagus, internal jugular vein, and carotid artery. In the thoracic spine, care must be taken to avoid the pleural cavity, thoracic aorta, and superior vena cava. In the lumbar spine, the abdominal aorta, inferior vena cava, renal vessels, nerve roots, and organs, such as, the kidneys should not be punctured.

**Figure 5:**
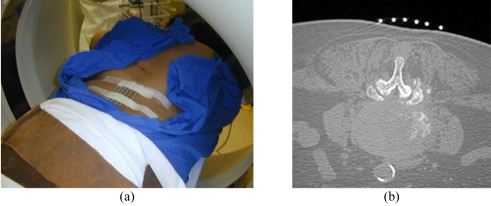
Grid method of surface marking for a lumbar biopsy. (a) Photograph shows placement of a grid of radio-opaque skin markers to help determine the needle entry point. (b) Preliminary axial CT image taken with the patient prone shows the grid of skin markers at the selected level. The most appropriate entry point can then be selected and surface-marked.

**Figure 6:**
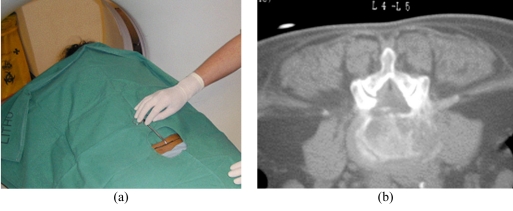
Free-hand method of surface marking for a lumbar biopsy. (a) Photograph shows the cleaned and draped patient lying in a prone position. A sponge forceps is used to indicate the estimated skin puncture point. (b) CT fluoroscopic image taken with the patient lying prone shows the tip of the sponge forceps at the proposed needle entry point. A right posterolateral paravertebral approach was planned for biopsy of the L4/5 disc.

The lesion depth, entry point, and angle of the chosen needle route should be estimated or measured on the most appropriate CT image. The point of entry at the patient’s skin is estimated or, alternatively, a grid of radio-opaque skin markers may be placed to help determine the entry point (Figure 5). The selected slice is surface marked, and the skin cleaned and draped (Figure 6). After administration of local anaesthesia (1% lignocaine), a small skin incision is made and the biopsy needle is directed into the lesion under intermittent CT fluoroscopy guidance. Some practitioners advocate infiltrating the paraspinal muscles and spinal periosteum as well with local anaesthetic, prior to insertion of the biopsy needle.

**Figure 7:**
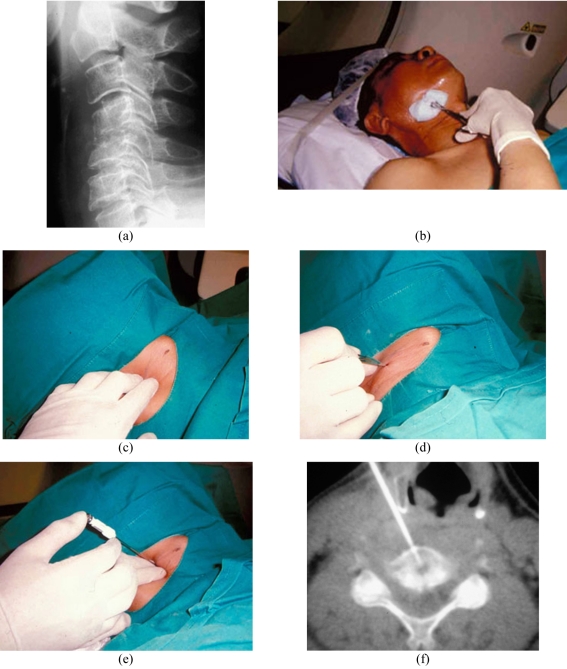
Biopsy of the C4/5 infective spondylodiscitis in a 50-year-old man using the anterolateral approach. (a) Lateral radiograph shows irregular narrowing of the C4/5 disc space with mild anterior wedging of C4 and C5 vertebral bodies and mild kyphotic deformity. (b) Photograph shows the patient lying in a supine position with the neck being cleaned after the selected level was surface-marked. (c) Photograph taken after the patient was draped shows the radiologist’s fingers palpating the carotid pulsation and guarding the right major neurovascular bundle. (d) Photograph shows the skin incision being made following administration of local anaesthetic. (e) Photograph shows insertion of the Ostycut biopsy needle. Note that the radiologist’s fingers are guarding the adjacent major neurovascular bundle during needle insertion. (f) CT fluoroscopic image taken with the patient lying supine shows the tip of the Ostycut needle within the inferior C4 vertebral body osteolytic lesion.

In biopsy of anteriorly-located lesions in the C3-C7 vertebrae, an anterolateral approach is usually adopted. As in the cervical discographic technique, the radiologists’ fingers are used to guard the carotid artery, together with the internal jugular vein and adjacent nerves, while the needle is directed towards the vertebral lesion. The needle should be inserted towards the vertebral body during the first pass, preferably from the patient’s right side, taking care to avoid puncturing the oesophagus and the trachea (Figure 7). Anteriorly-located lesions in C1 and the odontoid peg usually require a trans-oral approach, and in my opinion they should be preferably referred to the otolaryngological surgeon for biopsy.

**Figure 8:**
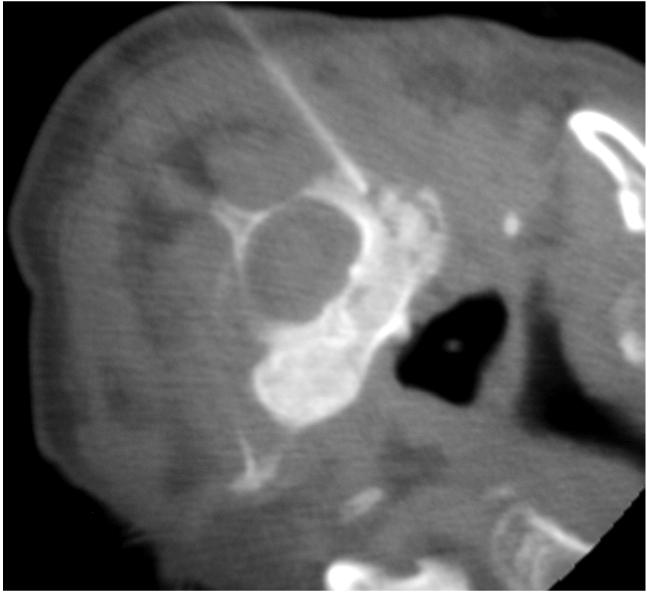
Biopsy of the C2 vertebra in a 61-year-old man with nasopharyngeal carcinoma complicated by C2 vertebral osteomyelitis. Axial CT fluoroscopic image taken with the patient in a lateral decubitus position shows the tip of the Ostycut needle within the right C2 lateral mass. A right posterolateral approach was used.

**Figure 9:**
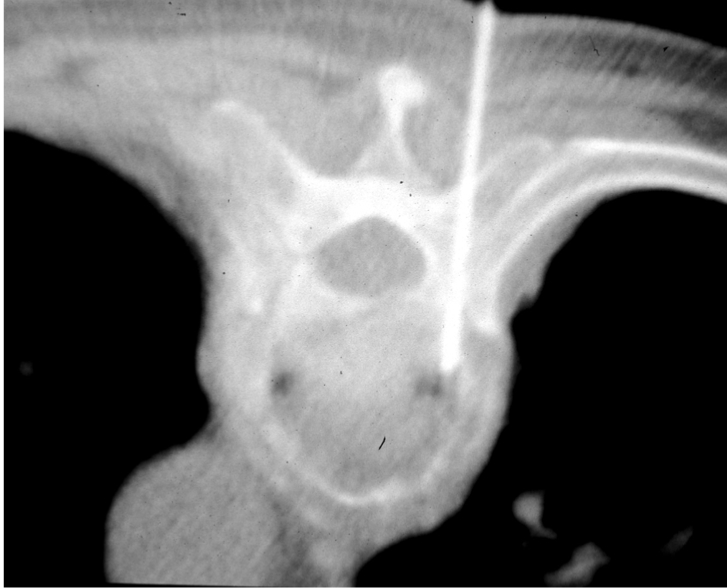
Biopsy of the T8 vertebra using the transpedicular approach in a 73-year-old man with metastasis. CT fluoroscopic image taken with the patient lying prone shows the tip of the Ostycut needle within the right side of T8 vertebral body.

**Figure 10:**
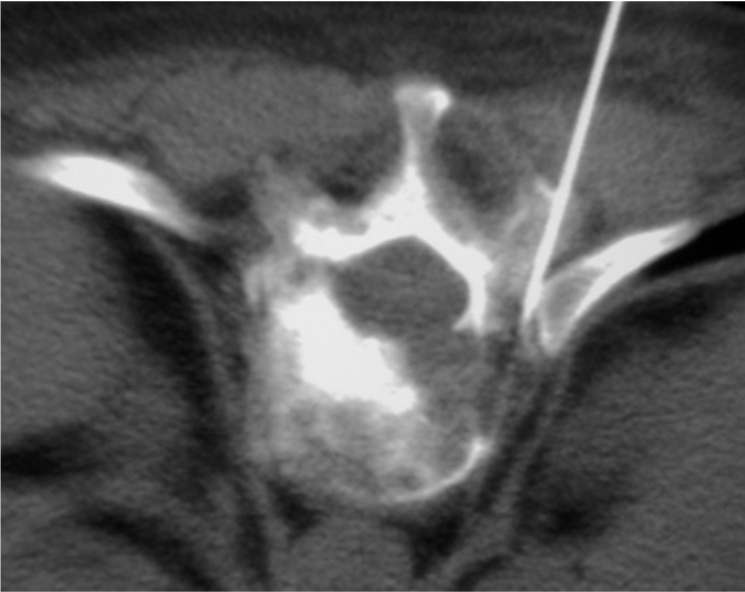
Biopsy of the T12 vertebra using the transcostovertebral approach in a 40-year-old woman with breast carcinoma. CT fluoroscopic image taken with the patient lying prone shows the tip of the Ostycut needle passing through the costovertebral junction towards the right T12 vertebral body osteolytic lesion.

**Figure 11:**
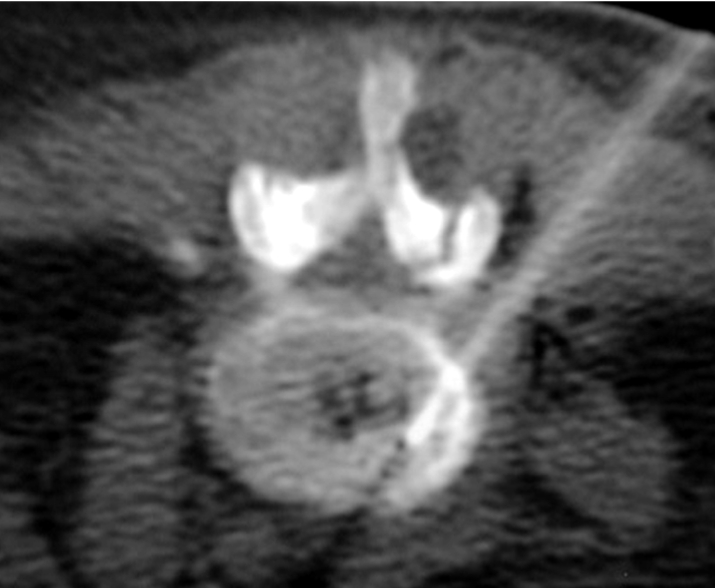
Biopsy of the L4/5 disc using the posterolateral paravertebral approach in a 66-year-old woman with infective discitis. CT fluoroscopic image taken with the patient lying prone shows the tip of the Ostycut needle within the right side of L4/5 disc. Note the streak artefact indicating the tip of the needle.

For posteriorly-located cervical spine lesions (Figure 8) and lesions in the thoracic and lumbar spine, the transpedicular (Figures 2 and 9), transcostovertebral (Figure 10), or posterolateral paravertebral (Figures 3, 6 and 11) approaches are usually used. The accuracy rates of biopsies using the transpedicular and posterolateral approaches are similar [3]. A lateral approach that enables access to the lumbar vertebral body, intervertebral disc, and paravertebral mass has been described. The patient lies in a lateral decubitus position within the CT scanner. In this position, the abdominal viscera are displaced forwards, providing a clear view of the lateral aspect of the lumbar spine for needle insertion. An advantage of this approach is that the needle tip is away from the nerve roots. The lateral approach should not be used, however, if forward displacement of abdominal contents is insufficient to safely avoid puncture of the abdominal viscera by the biopsy needle [11]. A transforaminodiscal approach has been reported to be a safe alternative to the posterolateral approach [15]. The approach to be adopted depends on the exact site of the lesion and should be appropriately tailored for each patient.

**Figure 12:**
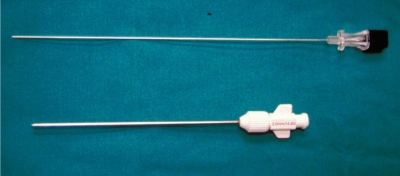
Photograph shows a Chiba aspiration needle (top) and an Ostycut trephine needle (bottom).

**Figure 13:**
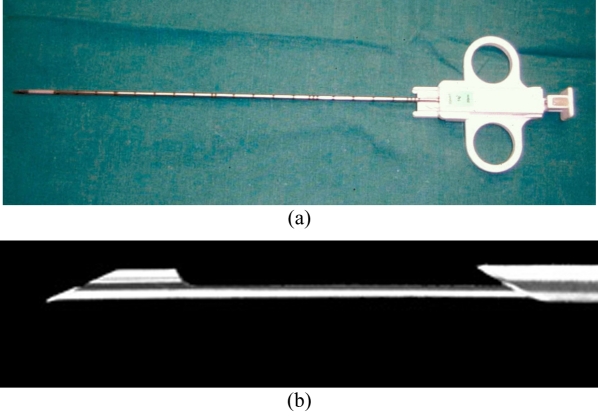
Quick-core cutting needle (Cook Medical Inc, Bloomington, IN, USA) used to obtain core biopsies of soft tissue. (a) Photograph shows the needle set that has a handle, which enables one-handed control and a spring-loaded trigger with a rapid-firing mechanism. (b) Close-up photograph of the needle tip shows the bevelled-point stylet that enables easy penetration into the lesion with minimal trauma to the surrounding tissue. Firing of the sharp cutting edge of the cannula facilitates obtaining an intact core tissue sample within the slotted stylet.

**Figure 14:**
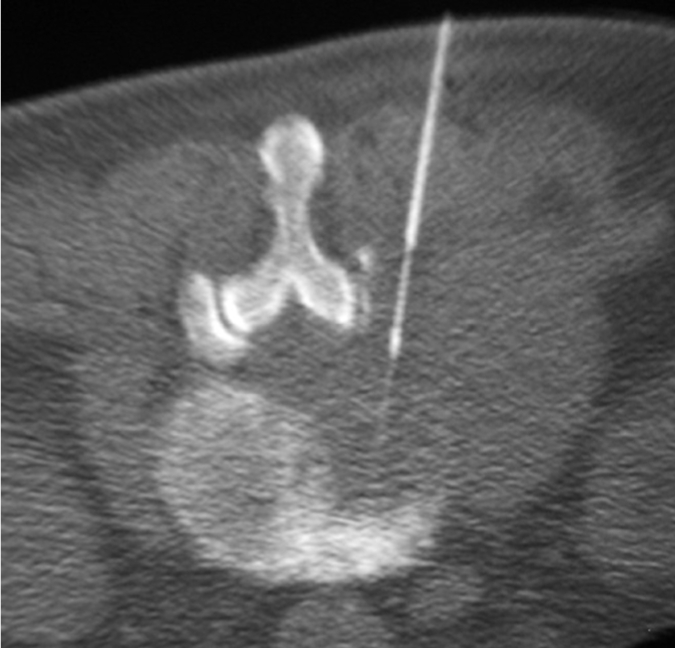
Use of a Temno cutting needle (Bauer Medical International, Santo Domingo, Dominican Republic) for biopsy of a large L4 vertebral lesion in a 79-year-old man with hepatocellular carcinoma. CT fluoroscopic image taken with the patient lying prone shows the tip of the cutting needle within the soft tissue component of the destructive osteolytic lesion that has extended into the right psoas muscle. Note that the bevelled-point stylet has been advanced in preparation for firing of the cutting cannula.

**Figure 15:**
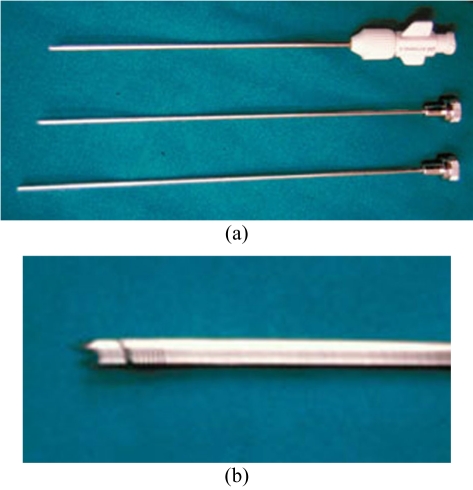
Ostycut trephine needle (Angiomed/Bard, Karlsruhe, Germany) used to obtain core specimens of bone. (a) Photograph shows the components of the needle set, comprising cannula (top), stylet (middle), and probe for dislodging the specimen at the end of the procedure (bottom). (b) Close-up photograph of the needle tip shows the threaded cannula and trocar-point stylet that facilitates penetration of cortical bone.

**Figure 16:**
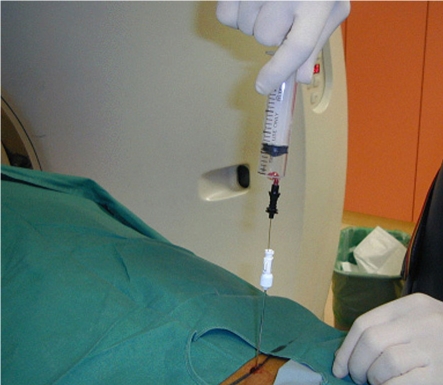
Coaxial technique in a patient undergoing lumbar spine biopsy. Photograph shows placement of a Chiba needle (black hub) within an Ostycut needle (white hub).

The type of needle used depends on the nature of the lesion and the operator’s personal preference. A large variety of needles are available commercially [44]. They can be broadly classified into aspiration (e.g. spinal or Chiba), cutting or tru-cut (e.g. Quick-core or Temno), and trephine (e.g. Ostycut, Craig or Ackerman) needles. They range in size from 11G to 22G. In general, the needle should be long enough to reach the lesion and have the appropriate bore size to obtain an adequate amount of specimen. Aspiration needles have a fine gauge and are best used to aspirate fluid, soft tissue lesions, or disc contents for culture or cytology (Figure 12). Cutting needles may be used for obtaining solid specimens from bone or, more usually, soft tissue (Figure 13). Both aspiration and cutting needles may also be used in bony lesions with overlying cortical destruction (Figure 14). Trephine needles have a serrated cutting edge and are usually required to obtain bone specimens for histopathology (Figure 15). A coaxial technique may also be used and decreases the need to re-puncture the patient as several samples may be obtained via a single tract [15] (Figure 16). Whichever needle type is used, CT fluoroscopy should be intermittently done to ensure that the needle tip is in a safe position during its insertion and to confirm its placement within the lesion.

**Figure 17:**
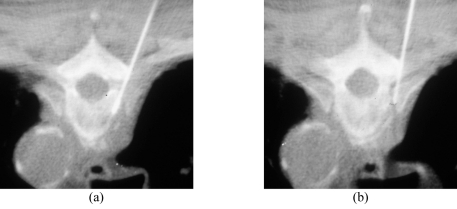
Multiple biopsies in a 61-year-old with T3/4 infective spondylodiscitis. CT fluoroscopic images taken with the patient lying prone show different placements of the needle tip to obtain specimens from both (a) bone and (b) soft tissue.

Ideally, the needle should be placed into different parts of the lesion to ensure representative sampling (Figure 17). In tumours, the necrotic or cystic areas should be avoided and the radiologist should try to identify these areas on CT or MR images prior to biopsy. Ideally, a cytotechnologist or cytopathologist should be present during the procedure to determine the adequacy of the tissue specimen. All material obtained should be sent for cytology, culture, and histopathological examination. CT-guided needle aspiration has been found to be accurate for identifying active bacterial disc infections, but is less reliable for fungal infections. Adding cytopathological analysis to microbiological analysis improves the sensitivity of infective lesions [12,19,45]. Blood clots may contain cells or organisms and should not be discarded [32]. Aspiration and core biopsy specimens have a complementary role [46].

**Figure 18:**
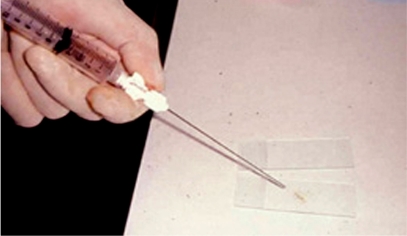
Photograph shows the biopsy specimen being smeared onto a glass slide for cytological examination.

**Figure 19:**
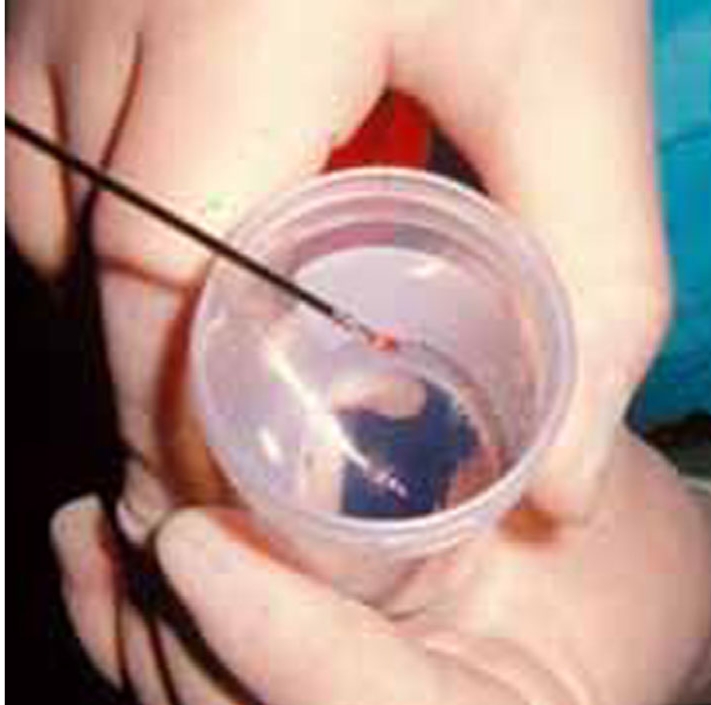
Photograph shows a bone core specimen being dislodged from the biopsy needle into a formaldehyde-filled container for histopathological examination.

**Figure 20:**
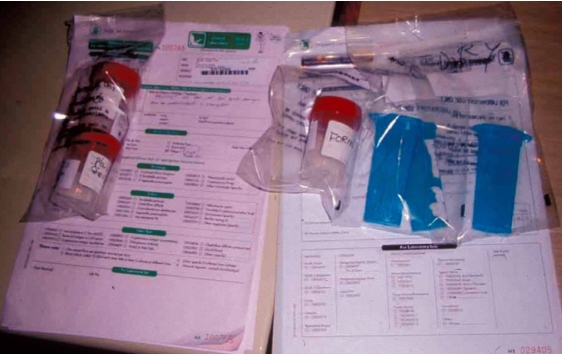
Photograph shows various containers, all of which are well-labelled, with accompanying request forms for the various tests.

The radiologist should be familiar with smear preparation on glass slides (Figure 18) and the various types of containers to be used for culture and histopathological specimens (Figure 19). All these specimens should be carefully labeled and dispatched promptly by a responsible person (Figure 20). The accuracy of the biopsy outcome depends on the type and location of the lesion, as well as the type of needle used. Core biopsy is more accurate than fine needle aspiration, while biopsies of malignant lesions have a higher accuracy rate compared with benign tumours and infection [7,13,18]. Compared with osteolytic malignant lesions, the diagnostic accuracy is decreased and the false-negative rate is higher for sclerotic lesions [17]. Thoracic level biopsies have been reported to have a lower diagnostic rate than lesions at other spinal levels [8].

After biopsy, the puncture site should be dressed and checked for possible complications. Prophylactic pain medication should be given to the patient. The length of the post- biopsy observation period depends on whether or not sedation was given. If the pleura is inadvertently punctured, further imaging to exclude a pneumothorax is indicated. The patient is sent home with instructions for pain medication, warned about possible complications, and told what to do if any occur.

## COMPLICATIONS

The types and incidence of complications depends on the type of needle used and on the anatomical location of the lesion. Reported incidence rates are 0 to 10%, with serious complication rates being less than 1% [1-4,6-8,10-11,15-18,21,28]. Risks for imaging- guided biopsy are acknowledged to be less than those associated with surgical open biopsy under general anaesthesia. The most frequently reported complications are pulmonary, neurological, and infective [1,16,17,40-43,46-49] and include:

- Bleeding requiring transfusion. Excessive local bleeding may be controlled by gelfoam insertion.
- Needle breakage.
- Infection.
- Neurological injury, including paresis or paralysis. Cord compression may rarely occur after biopsy of hypervascular spinal lesions, e.g., metastatic renal cell carcinoma or haemangioma.
- Pneumothorax. Rates of 4 to 11% after thoracic spine biopsy have been reported.
- Tumour seeding along the needle track.
- Infection spread along the needle track, with resultant formation of a draining sinus.

## CONCLUSION

CT-guided percutaneous biopsy has a useful role in the diagnosis and the management of patients with spinal lesions. Most biopsies can be safely and rapidly performed under local anaesthesia. Meticulous technique, knowledge of the spinal anatomy and of indications and contraindications, and awareness of possible complications are essential for success.
